# Chimps, Humans, and the Hominid Struggle against Viruses

**DOI:** 10.1371/journal.pbio.1002145

**Published:** 2015-05-28

**Authors:** Roland G. Roberts

**Affiliations:** Public Library of Science, Cambridge, United Kingdom

When a virus infects a cell in our bodies, rather than letting the incubus lie hidden, the cell displays tiny fragments of viral proteins on the cell surface. These peptide snips are neatly presented in a snugly fitting groove on molecular display stands called major histocompatibility complexes (MHCs). Here, roaming T cells from the immune system are free to probe the MHCs and see if their receptors recognize the presented peptides as being of foreign origin. In the case of class I MHCs, positive recognition by a passing cytotoxic T cell can result in the peptide-displaying cell (and its viral inhabitants) being targeted for destruction.

Because MHCs are in the front line of an arms race with the viral invaders, the genes that encode them tend to change rapidly during evolution in an attempt to keep one step ahead of their adversaries—the virus mutates to avoid recognition by the immune system, but the MHC mutates so that its groove can accommodate the altered viral peptide, and thus the cycle repeats. As a result, the MHC genes tend to vary considerably between individuals (hence the name MHC, which reflects their role in restricting our ability to transplant organs freely between unrelated people), and natural selection tends to favor populations with a high diversity of MHC genes.

The human immunodeficiency virus (HIV) is one virus that engages in such an arms race, and it’s known that one of the strongest genetic determinants of how HIV infection progresses is which version of one particular MHC class I gene an individual has. This gene, HLA-B, is the most variable in the entire human genome, with thousands of known forms in existence. One particular version—HLA-B*57:01—is strongly associated with having a lower viral load of HIV and progressing more slowly to full-blown acquired immunodeficiency syndrome (AIDS). One reason for this protective effect is presumed to be that peptides from the HIV Gag protein fit snugly into the groove of the HLA-B*57:01 version of the MHC, where they can be spotted by cytotoxic T cell receptors. Attempting to evade recognition, the virus also consistently mutates in HIV-infected people who have HLA-B*57:01 in a way that comes at a cost to the virus’s ability to replicate itself, reducing viral load.

Humans have encountered HIV only recently, when it crossed the species barrier from chimpanzees about a century ago, and the prevalence of HIV in most human populations is relatively low; consequently, the evolutionary effects of this virus on our repertoire of HLA-B variants are barely perceptible. Moreover, HLA-B can’t be studied in conventional model mammals like mice because the rapid evolution of MHC genes means that there is no direct equivalent of HLA-B in the rodent genome. To overcome these issues, a new study just published in *PLOS Biology* by Emily Wroblewski, Peter Parham, and coauthors takes a fascinating look at a parallel arms race taking place in our closest cousins.

The authors looked at wild Tanzanian chimps in Gombe National Park. These populations were made famous by the primatologist Jane Goodall and have been studied intensively for the last 55 years. Chimps live in social groups called communities, with females—but not males—tending to emigrate to a neighboring community when they mature. The Gombe chimps comprise three such communities, ranging in size from 11 to 62 individuals over the decades of observation, with a current total census of 99.

Although some work has been done on MHC diversity of captive chimps, this has been somewhat limited and has not yielded insights into natural population dynamics. The Gombe chimps have an additional feature that makes them a naturally occurring experiment: the endemic presence of simian immunodeficiency virus (SIV). Even more conveniently, the communities differ in the prevalence of SIV, with less than one-sixth of the northern and central communities infected but almost half of the southern community carrying the virus.

Collecting feces allowed the authors to glean a large amount of information on the animals with a minimum amount of disturbance—extraction of the fragmentary fecal DNA allowed them to identify the sex of individuals and the parental relationships between them and to characterize the MHC gene variants that they carry.

The authors chose to analyze two sections of the Patr-B gene—the chimp equivalent of human HLA-B. These two sections encode regions of the Patr-B protein that flank the peptide groove and determine which viral fragments will fit (Fig 1). Obtaining this information from the Gombe chimps enabled the authors to compare MHC diversity in four very different types of hominid populations: urban humans, indigenous tribal humans, the Gombe wild chimps (subspecies *Pan troglodytes schweinfurthii*, in which SIV is endemic), and a captive population of chimps in the Netherlands (subspecies *P*. *troglodytes verus*, which do not have SIV infection in the wild).

**Fig 1:**
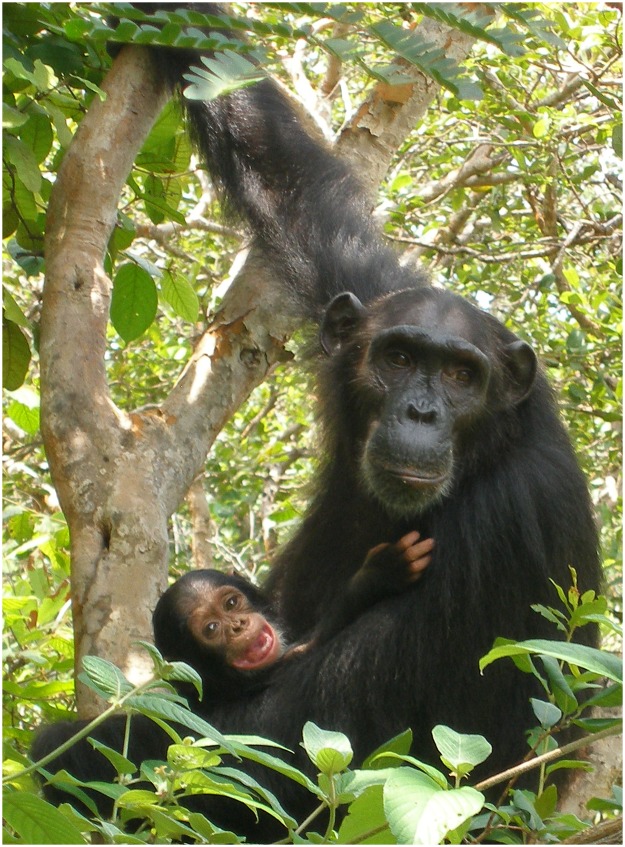
Fecundity has driven a few Patr-B variants to high frequency in the northern and central communities of wild chimpanzees in Gombe National Park, Tanzania. Image credit: Emily Wroblewski.

Despite their small population size, the Gombe chimps had 11 forms of Patr-B, most of which were previously undescribed. Moreover, these differed in distribution from neutral genetic markers like microsatellites and had a higher-than-expected tendency to affect the protein sequence—both characteristics that suggested selective forces might be generating and maintaining MHC diversity. These forces appear to be very different between the Gombe chimps and the captive ones, and the contrasting exposure to SIV is an obvious smoking gun.

Indeed, three forms of Patr-B are significantly more prevalent in SIV-infected chimps; perhaps the most interesting of these is Patr-B*06:03, a subtype of Patr-B that is only seen in the Gombe chimps and is overrepresented in SIV-carrying individuals. The authors show that Patr-B*06:03 lies in an unusual branch of the MHC family tree, a branch whose roots pre-date the separation of human and chimpanzee species and whose leaves include not only chimp Patr-B*06:03 but also several gorilla Gogo-B variants and human HLA-B*57:01. This latter, you may recall, is strongly associated with human responses to HIV.

The characteristic protein sequence features that define this trans-species group of MHC molecules all lie within one small region of the peptide grove, forming a pocket that accommodates the side chain of the second amino acid of the viral peptide. The specific sequences present in the Patr-B*06–03 and HLA-B*57–01 pocket suggest that they are adapted to bind to a serine or threonine at that position in the peptide.

An obvious conclusion is that, as with human HLA-B*57:01, this MHC variant might help the Patr-B*06:03-bearing chimps to manage their SIV infection better, survive longer, and thereby be overrepresented in the virally infected sample of the population.

Having the fecal samples allowed the authors to test this by amplifying SIV RNA from the feces as a means of estimating viral load. Their results suggest a substantially lower viral load in chimps that carry the Patr-B*06:03 version of the gene, consistent with the effects of the related HLA-B variant in humans.

The availability of historical samples meant that the authors could also examine the population dynamics of the Gombe chimps over a 15-year period. This showed that while the northern and central communities were relatively stable, the SIV-stricken southern community experienced pronounced movement of females both inward and outward, transforming its Patr-B repertoire. In the largest central community, a small number of socially high-ranking, fecund, and non-SIV-infected individuals dominated the production of new family members, feeding through to a high prevalence of two specific Patr-B variants.

Overall the study suggests how internal social and reproductive factors can combine with extraneous pathogens to influence the diversity of MHCs in this wild population of our closest relatives. It also paints a striking picture of a beleaguered community in a state of genetic flux, both from female emigration and from erosion by retroviral infection. With almost half of its members carrying SIV, the southern community has seen sweeping changes in its MHC repertoire in an evolutionary blink of an eye.

More intriguingly, the sustained presence of genetically and functionally equivalent MHC molecules in both humans and chimps (and quite possibly in gorillas) implies that despite the relatively recent origins of HIV and SIV, hominids may have been battling related lentiviruses for the best part of 10 million years.

## Abbreviations

AIDS: acquired immunodeficiency syndrome
HIV: human immunodeficiency virus
MHC: major histocompatibility complex
SIV: simian immunodeficiency virus
